# PREPARING NORTH CAROLINA’S HOME AND COMMUNITY-BASED SERVICES WORKFORCE: CURRENT CHALLENGES AND THE PATH FORWARD

**DOI:** 10.1093/geroni/igae098.1048

**Published:** 2024-12-31

**Authors:** Zavera Basrai, Caroline Yoon, Kezia Scales, Trish Farnham, Erin Carson, Nathan Boucher

**Affiliations:** Duke University, North Carolina, United States; Duke University, Durham, North Carolina, United States; PHI, New York, New York, United States; NC Coalition on Aging, Raleigh, North Carolina, United States; National Domestic Workers Alliance, Durham, North Carolina, United States; Duke University, Durham, North Carolina, United States

## Abstract

Older adults and people living with disabilities receive home- and community-based services (HCBS) from approximately 113,000 often poorly paid and inadequately supported direct service workers in North Carolina (NC). The demand for these workers is increasing rapidly, with NC projected to add almost 23,000 new direct care jobs from 2020 to 2030. The Medicaid-funded Workforce Engagement with Care workers to Assist, Recognize, and Educate (WECARE) project aims to modernize NC’s direct care training and credentialing (TC) to increase the pipeline of workers, build career pathways, elevate their skills and recognition, and improve job quality and retention using Collective Impact (CI). CI utilizes the following essential elements in creating and sustaining diverse collaborations: a common agenda, shared measurement, mutually reinforcing activities, continuous communication, and “backbone” support (dedicated coordination team). Informed by extensive desk research, five listening sessions with HCBS workers, support recipients, and family caregivers, and 113 formal engagements with state and non-profit agency leaders and regulatory bodies, WECARE’s state-wide landscape analysis revealed: lack of consistency and flexibility in TC requirements; limited portability of credentials; limited person-specific training; minimal funding incentives for professional development; and the need for an expanded core competency set, including planning, self-care, cultural humility and role delineation. To address these challenges, WECARE’s recommendations include adopting a core competency set for DSWs established through creating a unified multi-level DSW certification framework, strengthening career ladders and lattices within HCBS to support retention, and creating an online portal to house training, credentials, experience and other information that supports worker mobility.

